# Serrapeptase Eliminates *Escherichia coli* Biofilms by Targeting Curli Fibers, Lipopolysaccharides, and Phosphate Metabolism

**DOI:** 10.3390/microorganisms13081875

**Published:** 2025-08-11

**Authors:** Georgios Katsipis, Michalis Aivaliotis, Anastasia A. Pantazaki

**Affiliations:** 1 Laboratory of Biochemistry, Department of Chemistry, Aristotle University of Thessaloniki, 54124 Thessaloniki, Greece; gkatsipis@chem.auth.gr; 2 Laboratory of Neurodegenerative Diseases (LND), Center for Interdisciplinary Research and Innovation (CIRI), Aristotle University of Thessaloniki, 57001 Thessaloniki, Greece; 3 Laboratory of Biological Chemistry, School of Medicine, Faculty of Health Sciences, Aristotle University of Thessaloniki, 54124 Thessaloniki, Greece; aivaliotis@auth.gr; 4 Functional Proteomics and Systems Biology (FunPATh), Center for Interdisciplinary Research and Innovation (CIRI), Aristotle University of Thessaloniki, 57001 Thessaloniki, Greece; 5 Basic and Translational Research Unit, Special Unit for Biomedical Research and Education, School of Medicine, Aristotle University of Thessaloniki, 54124 Thessaloniki, Greece

**Keywords:** *Escherichia coli*, biofilms, serrapeptase, bacterial amyloids, virulence factors, phosphate metabolism

## Abstract

*Escherichia coli* biofilms are implicated in the development of persistent infections and increased antibiotic resistance, posing a significant challenge in clinical settings. These biofilms enhance bacterial survival by forming protective extracellular matrices, rendering conventional treatments less effective. Serrapeptase (SPT), a proteolytic enzyme, has emerged as a potential anti-biofilm agent due to its ability to degrade biofilm components and disrupt bacterial adhesion. In this study, we report the inhibitory effect of SPT against *E. coli* biofilm and its effect on key virulence factors. In vitro assays, including crystal violet staining, optical and fluorescence microscopy, and viability measurements, revealed the dose-dependent inhibition of biofilm formation (IC_50_ = 14.2 ng/mL), reduced biofilm (−92%, 500 ng/mL) and planktonic viability (−45%, 500 ng/mL), and a marked loss of amyloid curli fibers. SPT treatment also lowered the levels of key virulence factors: cellular and secreted lipopolysaccharides (−76%, 8 ng/mL; −94%, 32 ng/mL), flagellin (−63%, 8 ng/mL), and peptidoglycan (−29%, 125 ng/mL). Mechanistically, SPT induced a phosphate-dysregulating response: secreted alkaline phosphatase activity rose (+70%, 125 ng/mL) while cellular DING/PstS proteins declined (−84%, 64 ng/mL), correlating strongly with biofilm inhibition. In silico docking further suggests direct interactions between SPT and the curli subunits CsgA and CsgB, potentially blocking fiber polymerization. Together, these findings position SPT as a powerful non-antibiotic biofilm disruptor against *E. coli*, offering a promising strategy to undermine bacterial persistence and resistance by targeting both structural matrix components and metabolic regulatory pathways.

## 1. Introduction

*Escherichia coli* is a facultative anaerobic bacterial species. Regarded as the model Gram-negative bacterium, these bacteria can be universally found in the intestines of warm-blooded animals [1]. Despite this, some strains (including enterotoxigenic and enterohemorrhagic strains) are significant foodborne pathogens that may infest water bodies, fruits, vegetables, raw milk, or fresh meat [2]. These pathogenic strains or serotypes are the cause of a variety of human diseases, like hemolytic uremic syndrome and hemorrhagic colitis, leading to an estimated 2 million annual deaths worldwide [3]. In addition, *E. coli* strains can cause extraintestinal, life-threatening infections, such as those associated with medical devices, including but not limited to infections on urethral and intravascular catheters, prosthetic joints, shunts, and grafts [4]. Significantly, resistant *E. coli* strains are an alarming situation for medicine due to the overuse of antimicrobials that has generated tremendous genetic pressure on targeted pathogens but also on commensal bacteria [5].

Biofilms, which are considered the most widespread microbial ecosystems, are static growth formations surrounded by a protective layer of extracellular polymeric substance (EPS) [6]. EPS consists of a variety of biomolecules, including polysaccharides, proteins, lipids, and nucleic acids, which are crucial to the nutritional independence and hydration of the biofilm. Bacterial cells within the biofilm structure present differential properties when compared with their planktonic, freely growing counterparts. Significantly, biofilm-embedded bacteria are up to 1000 times more resistant to antibiotics, ultraviolet radiation, extreme temperature, pH, or salinity, as well as the host immune system, when compared with planktonic bacteria [7,8,9]. Recently, several studies have implied that biofilm-associated *E. coli* strains are significant carriers of multidrug-resistant (MDR) genes [9,10]. Moreover, biofilms pose a major industrial challenge by impairing performance, raising costs, and increasing environmental impact. In water treatment and cooling systems, biofilm accumulation on heat exchangers and pipelines reduces heat-transfer efficiency, elevates energy consumption, and accelerates material degradation [11]. In food processing, biofilm-associated microbes resist standard cleaning protocols, increasing contamination risks and costly product recalls [12].

Functional amyloids—a group of loosely ordered peptides—are regarded as crucial co-factors for the establishment and virulence of bacterial biofilms, as well as cell–cell interactions [13,14]. They are commonly found in the extracellular space, embedded in the EPS, and are recognized as long, unbranched fibers, composed of stacked β-sheet peptide formations. These peptides are highly resistant to protease treatment, routinely detected by their strong affinity with thioflavin T (ThT) and Congo red (CR) dyes [15,16]. An active metabolism is documented to be crucial for functional amyloid regulation and biofilm establishment [17]. The signature amyloid of *E.coli*, curli fibers, has been recognized for its ability to bind to both ThT and CR, with the peptide CsgA being the major fibril subunit and CsgB acting as the anchor seeding molecule [18]. In addition to curli, several bacterial virulence factors have also been implicated in differential, even serum-specific biofilm regulation, including Gram-negative bacterial endotoxin, namely lipopolysaccharides (LPSs) [19,20] and the flagellin subunit (FliC) of flagellum [21,22,23]. In addition, phosphate metabolism is intertwined with the biofilm formation mechanism, though the exact pathway is still under investigation [24,25,26,27,28].

Serrapeptase (SPT) is a serine protease that was first isolated from the Gram-negative bacterium *Serratia marcescens* [29]. Multiple reports have previously highlighted the possible therapeutic properties of SPT, with the most prominent being anti-inflammatory, analgesic, anti-amyloid, and anti-biofilm characteristics [30]. No significant side effects or toxicity were previously reported for in vivo or in vitro applications of SPT, thus promoting its possible pharmaceutical exploitation [31,32] and optimization of antibiotic therapy [33,34]. We have previously explored the anti-biofilm capacity of SPT against both susceptible and MDR *Staphylococcus aureus* strains and *Pseudomonas aeruginosa*, while also demonstrating its correlation with impaired bacterial viability, amyloid content, and virulence factors [26,28]. However, the effect of SPT on the significant pathogen *E. coli*, as well as its activity mechanisms, has not been studied until now.

Here, the anti-biofilm properties of SPT against *Escherichia coli* ATCC 25922 were assessed via the semi-quantitative crystal violet staining of mature biofilms and corroborated by light and fluorescence microscopy. To elucidate its impact on key virulence determinants, we measured LPSs and FliC expression using a dot blot. Concurrently, we evaluated the possible cytotoxic effect of SPT via viability assays, exploring its effect on phosphate metabolism. The capacity of SPT to disrupt functional amyloid formation within the biofilm matrix was probed by monitoring ThT and CR binding, while molecular docking simulations provided atomistic insights into SPT’s interactions with CsgA- and CsgB-derived amyloidogenic peptides. Finally, statistical analyses were performed to correlate alterations in virulence factor expression, amyloid disruption, and bacterial viability with biofilm inhibition, thereby delineating the mechanistic basis of SPT’s anti-biofilm activity. Though this is an initial “proof-of-concept” study, these findings pave the way for further in vivo validation and optimization of SPT as a therapeutic adjuvant, with the potential to combat recalcitrant biofilm-associated infections.

## 2. Materials and Methods

### 2.1. Chemicals and Bacterial Strain

SPT was received from capsules containing a total of 60,000 IU (Health Aid Ltd., Harrow, UK) and dissolved in 10 mL of growth medium. Before use, the solution was filtered through a Minisart NY 25, 0.45 μM filter (Sartorius Stedim Biotech GmbH, Goettingen, Germany) to remove non-dissolved material. No other purification was followed. The concentration of the SPT preparations was estimated with the Bradford assay, and the linearization of the standard curve, as suggested by Zor and Selinger [35,36]. The proteolytic activity of all solutions was verified prior to treatments, employing the chromogenic substrate azocasein (A-2765, Sigma-Aldrich, St. Louis, MO, USA) [37]. Tryptone (#403682), dimethyl-sulfoxide (DMSO) (#D5879), and (3-[4,5-dimethylthiazol-2-yl]-2,5-diphenyl tetrazolium bromide) (MTT) (#A2231) were acquired from PanReac AppliChem (Darmstadt, Germany). Para-nitro-phenyl-phosphate (pNPP) (#34081) was purchased from Cayman Chemical (Ann Arbor, MI, USA). Crystal violet (CV) (#C0775), CR (#75768), and thioflavin T (ThT) (#596200) were purchased from Sigma-Aldrich (St. Louis, MO, USA). Fluorescein diacetate (#1688) was purchased from Eastman Organic Chemicals (Kingsport, TN, USA). Propidium iodide (PI) (#40017) was from Biotium (Fremont, CA, USA). Sterile double-distilled H_2_O (sdd H_2_O) was used throughout the experimental procedures.

The *E. coli* strain Seattle 1946 (ATCC 25922) was employed for all microbiological studies.

### 2.2. Evaluation of Bacterial Growth and Biofilm Formation

All employed growth media were previously sterilized. Bacteria were stocked at −20 °C in a Luria–Bertani (LB) medium (% *w*/*v*: 1 g tryptone, 0.5 g NaCl, 0.5 g yeast extract), with 20% (*v*/*v*) glycerol. Initial cultures were performed by inoculating 10 mL of the LB medium with 100 μL of stocked culture, followed by overnight growth in a shaking incubator at 37 °C. Biofilm formation was performed by diluting bacterial pre-cultures 100 times with the biofilm medium [LB, 1% (*w*/*v*) D-glucose] for 24 h, at 37 °C, under unstirring conditions. The growth medium was standardized with pilot experiments and found to be the most suitable for providing quantitative biofilm formation and significant CR-positive amyloids under the current experimental conditions.

To estimate the growth of planktonic bacteria, the turbidity of the cultures, as determined by the absorbance at 630 nm, was read in a microplate reader (BioTek Instruments Inc., Winooski, VT, USA). To semi-quantify formed biofilms, *E. coli* was grown in polystyrene 96-well tissue-culture plates (TCPs) (#CLS3997, Corning, Corning, NY, USA) in the presence or absence of SPT. After 24 h of growth, media were discarded, and the wells were washed 2 times with PBS, dried for 30 min at 50 °C, and stained with CV (0.1%, *w*/*v*). Then, the dye was extracted from the biomass with 33% (*v*/*v*) acetic acid, and the solutions’ absorbance was determined at 570 nm. To test the ability of SPT to dissociate pre-formed biofilms, after biofilm formation, media were removed, residual planktonic cells were washed off twice with sterile PBS, and fresh biofilm medium was added containing increasing amounts of SPT. Then, bacteria were left for another 24 h at 37 °C under static conditions. The biofilm was then stained with CV and semi-quantified, as described above.

For staining and visualizing grown biofilms, 24-well TCPs (#CLS3526, Corning, Corning, NY, USA) were employed. The microscopy evaluation of CV-stained 24-well TCPs was performed after staining with 0,1% (*w*/*v*) CV for 10 min and observing the biofilm under standard light microscopy at 100× magnification (OCM 165, KERN & SOHN GmbH, Balingen-Frommern, Germany).

### 2.3. Determination of Planktonic Bacterial Viability by MTT Assay

*E. coli* bacteria were grown for 24 h in glass tubes sealed with a hydrophobic cotton gauze cap in the presence or absence of SPT and under biofilm conditions. Then, cells were received after centrifugation (5000 rpm, 10 min) and 3 subsequent washes with PBS and finally concentrated 20 times in sterile PBS. The viability of the bacterial cells was then determined after adding 10 μL of concentrated bacterial suspension to 190 μL of 0.5 mg/mL ΜΤΤ, and incubating at 37 °C, for 30 min, under shaking. Adequate negative controls were also run, containing only the PBS and the MTT substrate. After incubation, samples were centrifuged to formazan crystals, and DMSO was added to give a purple solution. Finally, absorbance was read at 570 nm in a microplate reader, and bacterial viability was determined after normalizing against bacterial growth and setting the control (untreated) culture at 100% viability [38].

### 2.4. Determination of Biofilm Bacterial Viability by Fluorescein Diacetate Assay

Fluorescein diacetate is a fluorogenic substrate that is hydrolyzed by a plethora of enzymes of metabolically active bacteria to produce fluorescein [39]. After *E. coli* biofilm growth in 96-well TCPs, 2 PBS washes were performed, and 100 μL of a 10 μg/mL solution of fluorescein diacetate in PBS was added to each well. A well without biofilm growth was used as a blank. The reaction was performed for 30 min at 37 °C with mild shaking in the dark. Then, the reaction buffer was transferred to another 96-well plate and was read in a Promega Glomax system (Madison, WI, USA) (exc. 490 nm; emis. 510–570 nm). Then, biofilm viability was estimated after subtracting the fluorescence of the blank and setting the value of the control sample as 100%.

### 2.5. Estimation of Amyloid and Peptidoglycan Content of Bacterial Surface

*E. coli* bacterial amyloids were determined with the CR assay, while peptidoglycan (PG) content was determined with CV staining after SPT treatment, as previously described [26,40]. Briefly, 10 μL of the concentrated bacteria suspension in PBS was mixed with CR or CV to receive a final dye concentration of 10 μg/mL. Samples without bacteria served as negative controls (the determination of the absorbance of the dye solutions), while samples with untreated bacteria served as positive controls. Bacteria were incubated at room temperature for 10 min with either CR or CV, collected by centrifugation (13,500 rpm, 15 min), and the absorbance of the received supernatant was read at 500 nm for CR and at 570 nm for CV. Finally, the bound dye corresponding to either bacterial amyloids or PG was estimated after subtracting the absorbance of the treated bacteria from the value of the negative control and normalizing before comparing this to the value of the positive control (set as 100%).

### 2.6. Fluorescence Microscopy of E. coli Biofilm Amyloids

Biofilms grown in 24-wells were washed twice with PBS, dried for 15 min at 50 °C, and fixed with 2.5% (*w*/*v*) glutaraldehyde for 15 min. Then, the biofilms were subsequently stained for 15 min with 10 μg/mL ThT for bacterial amyloids and 10 min with 1 μg/mL of PI for staining of whole biofilm mass (extracellular and genomic DNA), with two PBS washes following every staining step. Stained biofilms were then visualized and photographed at an inverted fluorescence microscope OCM 165 (ThT: exposure 120 ms, blue excitation; PI: exposure 60 ms, green excitation).

### 2.7. Determination of Intracellular and Extracellular Alkaline Phosphatase Activity

*E. coli* was treated with SPT and collected as previously described. For the determination of cellular alkaline phosphatase (ALP) activity, 50 μL of concentrated bacterial suspension was added to 450 μL of a 0.8 mM pNPP solution in a 100 mM Tris-HCl solution at pH 9.5, containing 100 mM NaCl and 5 mM MgCl_2_. Samples were then incubated at 37 °C for 20 min under mild shaking in the dark. The negative control sample contained only the chromogenic substrate. After incubation, the ALP reaction was terminated by the addition of 500 µL of 1 M NaOH, and samples were centrifuged to discard cells. Finally, the absorbance of the supernatant, corresponding to ALP activity, was read at 405 nm in a microplate reader. All results were normalized versus the bacterial turbidity at 630 nm.

For the determination of extracellular ALP activity, growth media were first collected and centrifuged at 13,500 rpm for 15 min at 4 °C. Samples were then lyophilized and reconstituted with H_2_O containing 1% (*v*/*v*) protease inhibitor to receive 20 times the concentrated media. To determine ALP activity, 50 μL of the concentrated media was mixed with 50 μL of ALP buffer, containing 1.6 mM pNPP, and incubated for 1 h at 37 °C under mild shaking in a 96-well microplate. The reaction was finally terminated with 100 μL NaOH, and the absorbance was read at 405 nm in a microplate reader and normalized compared to the total protein of the media, as determined by the Bradford-Bearden assay.

### 2.8. Lysis of Whole Bacterial Cells

Bacteria cultured in glass tubes, under biofilm conditions, were collected and resuspended at a final bacterial density of 2 OD (absorbance at 630 nm), in a 500 μL lysis buffer [100 mM Tris-HCl, pH 6.8, containing 10% *v*/*v* glycerol, 1% (*w*/*v*) sodium dodecyl sulfate, 2 mM ethylene-diamino-tetraacetic acid, 20 mM dithiothreitol, and 2 mM phenyl-methyl sulfonic acid]. The mix was freeze-thawed (−20 °C/4 °C) three subsequent times, followed by disruption via bead-beating with 0.5 mm glass beads (#Z250465, Sigma-Aldrich), employing 15 rounds (20 s), followed by 1 min on ice per cycle. The bacterial homogenate was finally centrifuged at 13,500 rpm for 15 min and 4 °C to remove debris and stored at −20 °C. The protein content of the lysates was determined with a BCA kit (#71285, Millipore, Darmstadt, Germany).

### 2.9. Determination of DING Proteins, FliC, and LPSs by Dot Blot

A dot blot immunoanalytical protocol was employed for the semi-quantification of cellular or secreted/extracellular DING proteins, the FliC subunit of bacterial flagella, and LPSs. DING proteins were detected with a rabbit polyclonal antibody (1:1000 dilution), prepared by GenScript (The Biology CRO) Company (Piscataway, NJ, USA), as previously described [41]. To detect FliC, a rabbit polyclonal antibody (#CSB-PA09734A0Rb) was employed (1:2000 dilution), purchased from Cusabio (Houston, TX, USA). For the determination of LPSs, a Hycult Biotech (Uden, The Netherlands) monoclonal mouse antibody (#HM6009) was employed against the lipid A compartment of LPSs (1:400 dilution). Secondary antibodies, including a goat anti-mouse IgG (#SA00002–1) and a goat anti-rabbit IgG (#SA00002–2), were conjugated with ALP at dilutions of 1:2000 and 1:4000, respectively (Proteintech, Manchester, UK). All antibody dilutions were performed with PBS containing 0.05% (*v*/*v*) Tween-20 (PBS-T).

The analysis was performed as previously described [26], by adding a drop of 5 μL of either bacterial lysate or concentrated medium on a 0.45 μΜ nitrocellulose membrane (71208, SERVA Electrophoresis GmbH, Heidelberg, Germany). After complete air drying, the membrane was blocked with 5% (*w*/*v*) skimmed milk for 30 min, and a diluted primary antibody was added for overnight incubation at 4 °C. After three subsequent washes with PBS-T, the membrane was incubated with the secondary antibody for 90 min. Following three more washes with PBS-T and one with the ALP buffer, the color development was performed for 30 min at 37 °C with 0.5 mM NBT and 0.5 mM BCIP. To semi-quantify the received dots, the Image J 1.49 (National Institutes of Health—NIH, Bethesda, MD, USA) application was used. All results were normalized compared to the protein content of the lysates.

### 2.10. Statistical Analyses

All statistical analyses and graphical representations were performed using GraphPad Prism version 8 (GraphPad Software Inc., San Diego, CA, USA). Each experiment was conducted using at least three independent biological replicates, and within each, a minimum of three technical replicates was included. The results are presented as mean values ± standard errors of the mean (SEM). To assess the differences between control and experimental groups, one-way ANOVA was applied using either Brown–Forsythe or Welch correction, depending on variance homogeneity. Dunnett’s post hoc test was used for comparisons to the control, following the confirmation of normal data distribution. Normality was verified using the Shapiro–Wilk test. The half-maximal inhibitory concentration (IC_50_ or EC_50_) and its 95% confidence interval (CI 95%) were determined from log-transformed dose–response curves, using the following equation: log [SPT] vs. normalized inhibition (%). This was fitted with a variable slope model. Correlation analyses were performed using Pearson’s *r* and two-tailed *p* values. Statistical significance was set at *p*  <  0.05. Significance levels are denoted as follows: *p*  <  0.05 (*), *p*  <  0.01 (**), *p*  <  0.001 (***), and *p*  <  0.0001 (****).

### 2.11. In Silico Analysis of the Interaction Between SPT and the Curli Subunits CsgA and CsgB

In silico analysis of the interaction between SPT and the curli subunits CsgA and CsgB was performed using AlphaFold Server [42]. The analysis was performed using the amino acid sequences of the proteins from the UniProt Database (https://academic.oup.com/nar/article/53/D1/D609/7902999?login=false, accessed on 19 June 2025), including one Zn^2+^ and seven Ca^2+^ metals known to bind in specific regions of the folded SPT protein. In addition to AlphaFold, the HADDOCK 2.4 Web Server was used to predict protein interactions and determine docking parameters and confidence metrics [43,44].

## 3. Results

### 3.1. SPT Inhibits the Biofilm Formation of E. coli

**Figure 1:**
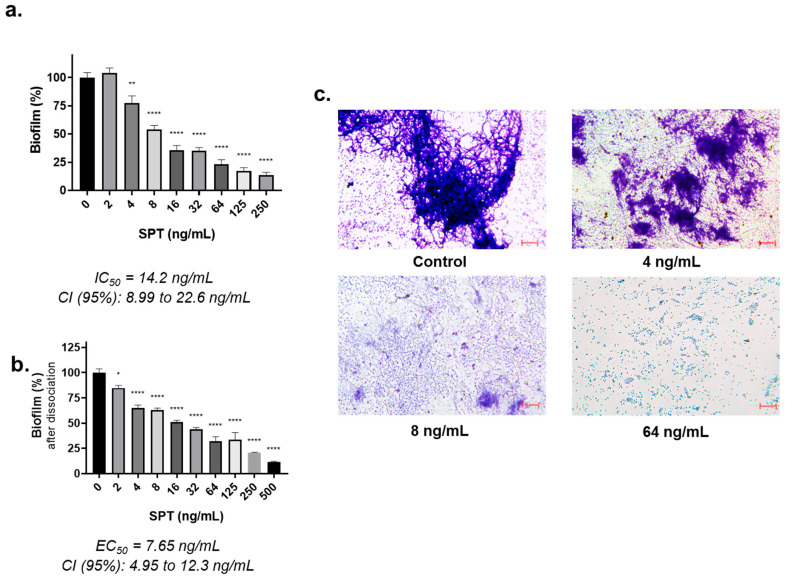
Inhibition of *Escherichia coli* ATCC 25922 biofilm formation by serrapeptase (SPT). *E. coli* was grown on a plastic surface in the presence or absence of SPT (control). Biofilm formation (**a**) is shown as determined by crystal violet staining after dye extraction. (**b**) The disaggregating effect of SPT on pre-formed biofilms was also verified. (**c**) Photographs of biofilms formed in plastic TCPs in the presence or absence of various concentrations of SPT. Scale bar: 100 μm. The bars in the graphs show mean values ± SEMs from at least three biological replicates, with the control value set at 100%. Statistical analysis was performed using one-way ANOVA followed by Dunnett’s post hoc test for the correction of multiple comparisons. Statistically significant differences between untreated controls and SPT-treated samples are indicated as follows: *p* < 0.05 (*), *p* < 0.01 (**), and *p* < 0.0001 (****).

The possible anti-biofilm activity of SPT against *E. coli* was studied in TCPs. After staining formed biofilms with CV, semi-quantification was performed after the extraction of the dye and spectrophotometric determination at 570 nm. The results of the analysis are provided in Figure 1a. SPT is verified as a strong inhibitor of biofilm formation of *E. coli* with an IC_50_ value of 14.2 ng/mL (CI 95%: 8.99 to 22.6 ng/mL). Maximum inhibition of biofilm formation was found at 0.25 μg/mL and was −87% (*p* < 0.0001) that of the control. In addition, the ability of SPT to disrupt performed biofilms of *E. coli* on TCPs was also studied, and the results are provided in Figure 1b. SPT treatment significantly detached the biofilm formations in a concentration-dependent manner, with an EC_50_ calculated at 7.65 ng/mL (CI 95%: 4.95 to 12.3 ng/mL).

Indicative photographs of *E. coli* biofilms stained with CV, in the presence or absence of SPT, are presented in Figure 1c. Control biofilms on plastic surfaces are characterized by elongated fibrous formations with embedded bacteria and some sparse bacterial aggregates. Under SPT treatment, fibrous formations were reduced to smaller aggregates and were eventually fully dispersed at the higher studied dosage of 64 ng/mL. At the highest concentration of SPT studied, biofilm formation was almost eliminated, in correlation with the results obtained from growth on TCP plastic surfaces.

### 3.2. SPT Treatment Impairs the Growth and Viability/Metabolic Activity of E. coli

**Figure 2:**
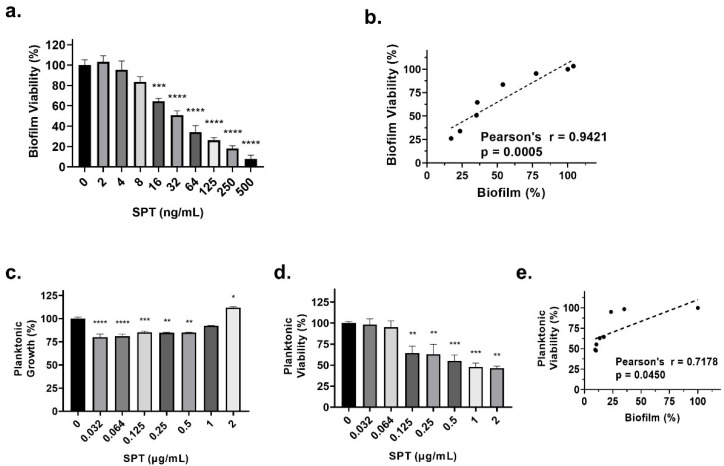
(**a**) *Escherichia coli* ATCC 25922 biofilm viability (%) after 24 h of treatment with serrapeptase (SPT) (0–500 ng/mL). (**b**) The positive correlation between residual biofilm biomass (%) and viability. (**c**) Planktonic growth (%) following 24 h of exposure to SPT (0.032–2 µg/mL). (**d**) Planktonic cell viability (%) under the same conditions. (**e**) The correlation between residual biofilm biomass (%) and planktonic viability. *E. coli* was grown on a plastic surface for the determination of the bacterial biofilm, and in glass tubes for the assessment of planktonic bacteria growth. Growth was determined by measuring the turbidity of the culture at 630 nm, while viability was determined with the MTT test. The bars in the graphs show the mean values ± SEMs from at least three biological replicates, with the control value set at 100%. Statistical analysis was performed using one-way ANOVA followed by Dunnett’s post hoc test for the correction of multiple comparisons. Statistically significant differences between untreated controls and SPT-treated samples are indicated as follows: *p* < 0.05 (*), *p* < 0.01 (**), *p* < 0.001 (***), and *p* < 0.0001 (****).

To estimate the possible effect of SPT on *E. coli* growth and viability, bacteria were grown under biofilm conditions for 24 h in the presence or absence of SPT. Then, the rough turbidity of the cultures was read at 630 nm, while the viability of either planktonic or biofilm-attached bacteria was determined with the MTT assay. The results of the determination of biofilm viability are provided in Figure 2a, while the planktonic bacteria results are shown in Figure 2d. The effect of SPT on planktonic bacterial growth is shown in Figure 2c.

The viability of *E. coli* biofilm was found to be disrupted by the effect of SPT in a dose-dependent manner (Figure 2a) with maximal inhibition verified at 500 ng/mL (−92%, *p* < 0.0001). As expected, biofilm viability is perfectly correlated to the inhibition of biofilm formation (Figure 2b), with the statistical analysis resulting in Pearson’s *r* = 0.9421 and a *p*-value of 0.0005. A significant decrease in the growth of *E. coli* bacteria was demonstrated for almost all SPT doses used in terms of culture turbidity (Figure 2c). A maximal reduction in *E. coli* growth was verified at 0.032 μg/mL SPT and was −20% that of the control culture (*p* < 0.0001). However, when increasing doses of SPT were employed, turbidity reductions were restricted, with a significant, slight increase at culture turbidity demonstrated at 2 μg/mL of SPT (*p* = 0.0133). A reduction in the viability of planktonic bacteria was also found (Figure 2d), with maximal inhibition verified at 2 μg/mL (−54%, *p* = 0.0015). Correlation analysis indicated that reduced bacterial growth does not coincide with biofilm inhibition. On the other hand, the reduction in viability was significantly correlated with biofilm inhibition (Pearson’s *r* = 0.7178, *p* = 0.045) (Figure 2e). These results demonstrate that SPT presence can impair *E. coli* viability, and this effect is strongly associated with the compound’s anti-biofilm potential.

### 3.3. The SPT Treatment Reduces Functional Amyloid and Peptidoglycan Titers of the E. coli Cell Wall

**Figure 3:**
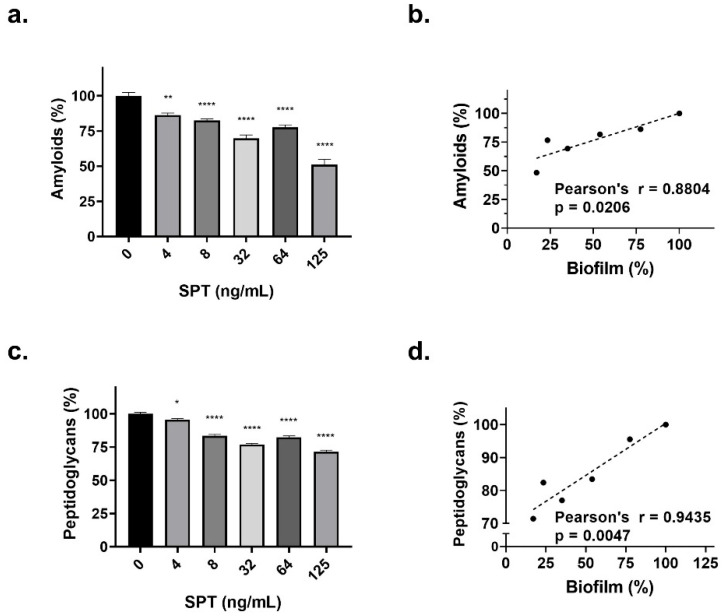
Reduction in (**a**) bacterial amyloids and (**c**) peptidoglycan titers of *Escherichia coli* ATCC 25922, after treatment with serrapeptase (SPT). *E. coli* was grown statically in glass tubes, and the levels of amyloids and peptidoglycan were determined after staining with Congo red or crystal violet, respectively. Correlation analyses were performed to evaluate the possible interrelation of (**b**) amyloids or (**d**) peptidoglycans levels with the residual biofilm mass (%). Correlations with *p* < 0.05 were regarded as statistically significant. The bars in the graphs represent mean values ± SEMs from at least three biological replicates, with the control value set at 100%. Statistical analysis was performed using one-way ANOVA followed by Dunnett’s post hoc test for the correction of multiple comparisons. Statistically significant differences between untreated controls and SPT-treated samples are indicated as follows: *p* < 0.05 (*), *p* < 0.01 (**), and *p* < 0.0001 (****).

The impact of SPT on *E. coli* curli fibers was assessed using the amyloid-binding dye CR. Bacterial cultures were incubated with CR, and the amount of dye retained—reflecting the functional amyloid content—was quantified spectrophotometrically. As shown in Figure 3a, SPT treatment produced a concentration-dependent decline in curli levels, with the greatest inhibition (~52% reduction relative to untreated controls) observed at 0.12 µg/mL (*p* < 0.0001). Furthermore, correlation analysis (Figure 3b) revealed a strong, positive association between the decrease in CR retention and the extent of biofilm suppression. These findings highlight the critical role of curli fibers in *E. coli* biofilm development and suggest that SPT’s anti-biofilm efficacy is at least partly mediated by its ability to disrupt amyloid assembly.

To evaluate the effect of SPT treatment on the *E. coli* bacteria cell wall, bacteria grown under biofilm conditions were stained with CV, which is a dye that adheres to the PG layer of the bacterial cell wall. The PG titers of SPT-treated and control cultures were then estimated inversely and spectrophotometrically with the retention method, and the normalized results are provided in Figure 3c. The results revealed that *E. coli* bacteria grown in the presence of SPT reduced levels of PGs, in comparison with control cultures, and SPT may disrupt the physiological structure of the cell wall, at least in terms of the PG content. The maximum reduction in PG titers was found at 0.12 μg/mL of SPT, which is −29% that of the control (*p* < 0.0001). In addition, correlation analysis for the possible interrelation of PG levels with biofilm formation due to the effect of SPT was conducted, the results of which are presented in Figure 3d. Analysis demonstrated that a reduction in the levels of PGs correlates in a statistically significant and positive manner with biofilm inhibition, indicating that alterations in the physiology of the bacterial cell wall may impair its ability to form biofilm, adding another perspective to the anti-biofilm efficacy of SPΤ.

**Figure 4:**
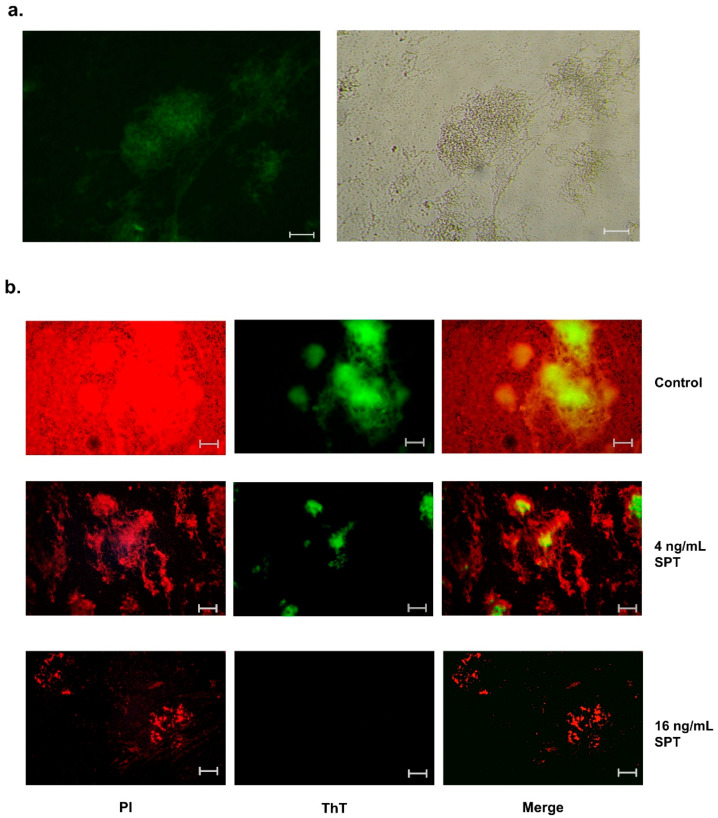
Fluorescence microscopy of thioflavin T (ThT)-positive bacterial amyloids in biofilms of *Escherichia coli* ATCC 25922 in the presence or absence of serrapeptase (SPT). *E. coli* was grown under static conditions in tissue culture plates (TCPs) for 24 h. (**a**) A representative image of a large amyloid-associated fibrous formation from an untreated (control) culture of *E. coli*, as photographed under light microscopy (**right**) and FITC-fluorescence microscopy (**left**). (**b**) The visualization of ThT-positive amyloid fibers in cultures treated with 4 or 16 ng/mL of SPT or untreated (control) ones. Propidium iodide (PI) was employed in all cases as a general stain for the biofilm mass. Merged figures reveal the distribution of bacterial amyloids in the biofilm mass. Scale bar: 100 μm.

To further elucidate the employment of bacterial amyloids in the biofilm structure, under the effect of SPT or in its absence, biofilms formed in TCPs were stained with ThT and visualized under a fluorescence microscope. Interestingly, the large fibrous mass found in *E. coli* biofilms (see also Figure 1c) coincides with amyloid staining (Figure 4a). The dissolution of these fibrous formations and reduction in the biofilm mass (PI fluorescence) under the effect of 4 or 16 ng/mL of SPT is associated with a significant drop in ThT fluorescence (Figure 4b). These observations further correlate with the interrelation between biofilm breakage and the functional amyloids of *E. coli*.

### 3.4. SPT Treatment Alters the Phosphate Metabolism of E. coli

**Figure 5:**
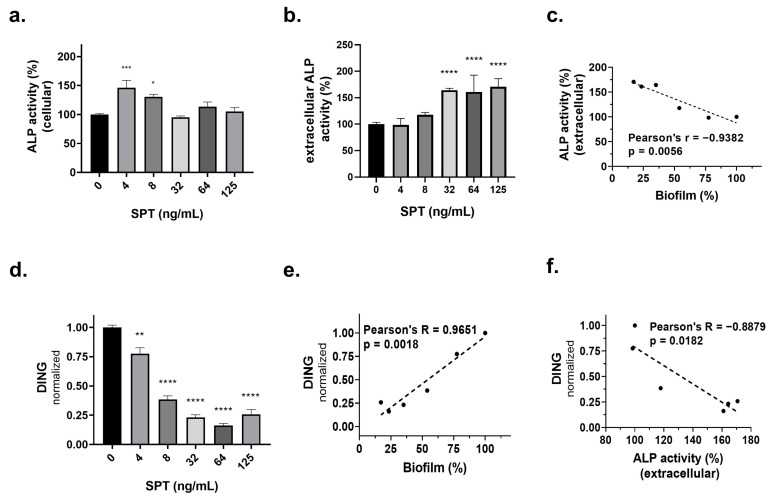
The effect of several concentrations of SPT on the (**a**) cellular and (**b**) extracellular activity of alkaline phosphatase (ALP) activity and (**d**) levels of DING proteins from *Escherichia coli* ATCC 25922. *E. coli* was grown under static conditions in glass tubes, and ALP activity was determined after hydrolysis of the pNPP substrate and subsequent reads at 405 nm. For DING levels, bacteria were homogenized and lysates were analyzed with dot blot, employing an antibody against the sequence DINGGGATLPQPLYC of the phosphate ABC transporter periplasmic protein. Correlation analyses were performed to evaluate the possible implication of the (**c**) extracellular activity of ALP or (**e**) DING levels on the inhibition of biofilm formation. (**f**) The correlation of DING levels with extracellular ALP activity. Correlations with *p* < 0.05 were regarded as statistically significant. The bars in the graphs represent mean values ± SEMs from at least three biological replicates, with the control value set at 100%. Statistical analysis was performed using one-way ANOVA followed by Dunnett’s post hoc test for the correction of multiple comparisons. Statistically significant differences between untreated controls and SPT-treated samples are indicated as follows: *p* < 0.05 (*), *p* < 0.01 (**), *p* < 0.001 (***), and *p* < 0.0001 (****).

As phosphate is a core component of bacterial metabolism, the effect of SPT treatment on the activity of cellular and secreted ALP of *E. coli* has been studied. The results for cellular and extracellular ALP activity are depicted in Figure 5a and Figure 5b, respectively. The cellular ALP activity of *E. coli* cells significantly increased at the lower employed dosage of SPT (0.004 and 0.008 μg/mL). On the other hand, the secreted ALP activity, as determined in the culture media, significantly increased at higher employed SPT concentrations (0.03–0.12 μg/mL). The maximum increase in cellular ALP activity was found at 0.004 μg/mL of SPT and was 146% that of the control (*p* = 0.0004), while the maximum increase in secreted ALP activity was found at 0.12 μg/mL of SPT compared to that of the control at 171% (*p* = 0.0005). Correlation analysis was also performed to investigate a possible interrelation with SPT-induced biofilm inhibition, and the respective graph is presented in Figure 5c. Indeed, a strong negative correlation was found between the secreted ALP activity and the biofilm titers of *E. coli*, implying that an increase in extracellular ALP levels coincides with the disintegration of the bacterial biofilm.

In addition, the levels of DING proteins, which are crucial effectors of phosphate homeostatic mechanisms, were also determined, and the results are presented in Figure 5d. SPT treatment led to a dose-dependent decrease in DING protein levels, with maximal inhibition found at 0.006 μg/mL of SPT, which was −84% that of the control (*p* < 0.0001). Correlation analysis proved that the decrease in the levels of the DING protein is significant in accordance with decreased biofilm formation (positive correlation, Figure 5e) and the increased extracellular activity of ALP (negative correlation, Figure 5f). These results underline the implication of the phosphate metabolism in the life cycle of *E. coli* biofilm.

### 3.5. SPT Treatment Leads to a Reduction in Crucial Virulence Factors, Such as LPSs and FliC

**Figure 6:**
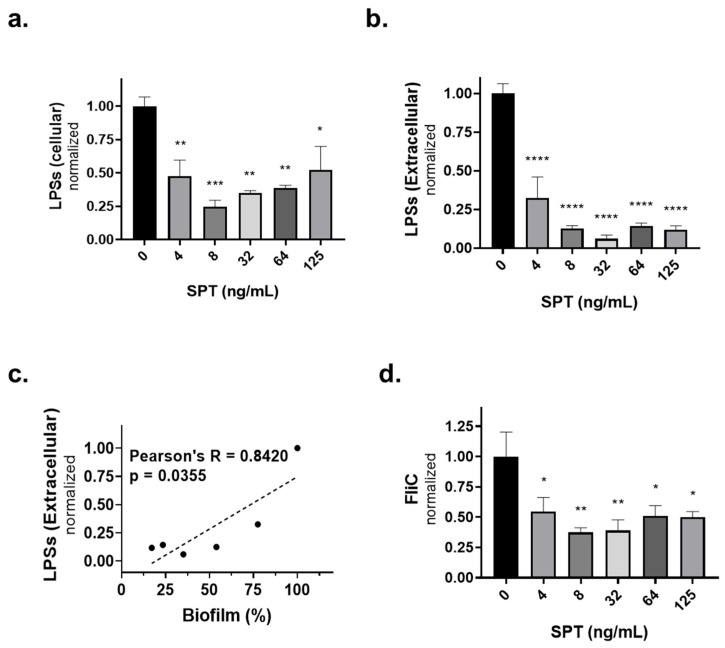
The effect of several concentrations of SPT on (**a**) the cellular and (**b**) extracellular levels of lipopolysaccharides (LPSs) and (**d**) levels of flagellin subunit C (FliC) protein from *Escherichia coli* ATCC 25922. *E. coli* was grown under static conditions in glass tubes; bacteria were homogenized; and lysates were analyzed with a dot blot, employing an antibody against either LPSs or FliC. (**c**) Correlation analysis of secreted LPS levels with the inhibition of biofilm formation. Correlations with *p* < 0.05 were regarded as statistically significant. The bars in the graphs show mean values ± SEMs from at least three biological replicates, with the control value set at 100%. Statistical analysis was performed using one-way ANOVA followed by Dunnett’s post hoc test for the correction of multiple comparisons. Statistically significant differences between untreated controls and SPT-treated samples are indicated as follows: *p* < 0.05 (*), *p* < 0.01 (**), *p* < 0.001 (***), and *p* < 0.0001 (****).

To investigate whether the SPT treatment of *E. coli* can restrict crucial bacterial virulence factors, the levels of endotoxins, LPSs, and the flagellar component FliC protein were investigated. After bacteria were grown under biofilm conditions, cells were lysed, and LPSs and FliC were detected with a dot blot assay. In addition, dot blot analysis was performed at concentrated growth media to detect extracellular LPSs and FliC. Figure 6a,b present the cellular and extracellular levels, respectively, of LPSs. SPT treatment led to a dose-dependent decrease in both cellular and extracellular LPS titers; the maximum inhibition of cellular LPSs was verified at 0.008 μg/mL of SPT (−75% to that of the control, *p* = 0.0004), and the maximum inhibition of extracellular LPSs was found at 0.03 μg/mL of SPT (−94% to that of control, *p* < 0.0001). Correlation analysis proved that the amelioration of extracellular LPS levels under the effect of SPT is positively interrelated with biofilm inhibition (Figure 6c), while such an effect was not verified for cellular LPS levels. As LPSs are the main endotoxins implicated in *E. coli* infections and biofilm formation, these results further promote SPT as a promising antibacterial agent.

Regarding the FliC protein, SPT treatment also caused a significant, dose-dependent decrease in its cellular levels (Figure 6d), while extracellular levels could not be detected. This result was expected, as this protein’s levels are probably very low, below the quantitation limit of our employed methodology, as FliC is not a standard secreted factor like LPS endotoxins. The maximum decrease in cellular FliC levels was documented with a dose of 0.008 μg/mL of SPT (−63%, *p* = 0.0071). Correlation analysis between the levels of FliC and biofilm inhibition using SPT did not return any significant results. However, the strong decrease in FliC levels via SPT treatment reveals a possible link between this flagellar component and the physiology of the *E. coli* biofilm, or even the direct proteolytic effect of SPT.

### 3.6. SPT Is In Silico Predicted to Interact with Curli Subunits CsgA and CsgB

**Figure 7:**
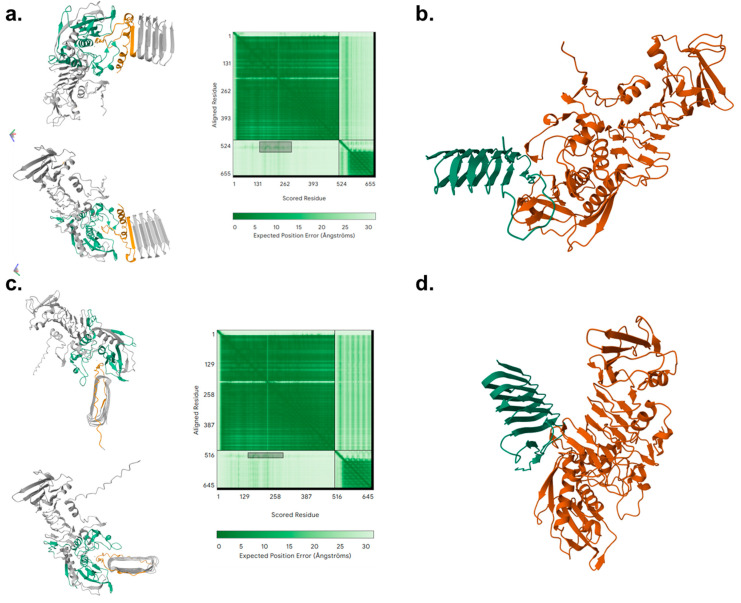
Molecular docking of serrapeptase on curli subunits CsgA (**a**,**b**) and CsgB (**c**,**d**). Protein structure predictions for the CsgA and CsgB subunits were generated using the AlphaFold server, and docking simulations with serrapeptase were performed using the HADDOCK 2.4 server. Panels (**a**,**c**) (**left**) show the AlphaFold-predicted three-dimensional structures of CsgA and CsgB, respectively, in complex with serrapeptase, highlighting the predicted binding interface. In these structural models, serrapeptase is shown in **orange**, while the curli subunits are depicted in **green** (binding-region) and **grey** (remaining protein). Panels (**a**,**c**) (**right**) display the corresponding Predicted Aligned Error (PAE) plots from AlphaFold, where darker green indicates lower expected positional error (higher confidence) and lighter shades indicate greater uncertainty. Panels (**b**,**d**) present the top-scoring docking conformations obtained from HADDOCK, with serrapeptase in **orange** and the curli subunit in **green** for clarity of interaction site visualization.

The in silico analysis of interactions between SPT and Csg subunits A and B confirmed the interaction of SPT with both subunits in the same region of SPT (Figure 7). AlphaFold analysis (Figure 7a,c) indicated that the interface of interactions with subunits A and B (green color) is estimated to involve amino acid residues of SPT 147–158 and 214–207. The amino acid residues involved in the interphase of interactions in subunit A are 14–44, and the amino acids involved in subunit B are 18–30, indicated with an orange color.

HADDOCK clustered 190 structures in seven clusters, which represent 95% of water-refined models that are HADDOCK-generated. The top cluster is the most reliable according to HADDOCK (Figure 7b,d). The HADDOCK docking analysis revealed that serrapeptase forms stable complexes with both curli peptides CsgA and CsgB.

CsgA–SPT

The top-ranked cluster (41 models) scored −146.5 ± 7.2 (HADDOCK score) with a Root Mean Square Deviation (RMSD) of 0.4 ± 0.3 Å and a large, buried surface area of ~2623 Å^2^. Major energetic contributions arose from van der Waals interactions (−95.9 ± 2.6 kcal·mol^−1^) and electrostatic forces (−138.8 ± 27.2 kcal·mol^−1^), while the desolvation energy was −39.1 ± 2.4 kcal·mol^−1^. Key contacts involve the following complementary charged residues: basic side chains (Arg, Lys) on CsgA engage acidic/polar residues on serralysin, underpinning the complex’s stability.

CsgB–SPT

The leading cluster (115 models) scored −85.4 ± 2.4 kcal·mol^−1^ with an RMSD of 0.8 ± 0.6 Å and a buried surface area of ~2123 Å^2^. Electrostatic interactions dominated (−174.9 ± 11.5 kcal·mol^−1^) with significant van der Waals contributions (−72.5 ± 4.2 kcal·mol^−1^) and minimal desolvation penalty (−0.6 ± 1.7 kcal·mol^−1^), indicating that charged contacts drive complex formation.

The predicted complex models from AlphaFold showed consistent interface geometry with a low expected position error at the contacting residues, independently validating the docking-derived binding mode.

## 4. Discussion

Biofilms are not only the most dominant bacterial ecosystem, but also a way for building MDR [10] and chronic infestation of human tissues and prosthetics [45,46]. *E. coli* is one of the core human pathogens, and its biofilm formation was previously correlated with increased antibiotic resistance [47]. In addition, the overuse of antibiotics is now openly framed as one of the core perpetrators of MDR gaining [9], and the employment of non-antibiotic agents against bacterial infections is urgently needed. Thus, in the current study, the employment of SPT against *E. coli* biofilms was studied.

SPT has been shown to both prevent and disrupt biofilms formed by various bacterial species in both in vitro and in vivo settings, including *Listeria monocytogenes* [48], *Staphylococcus aureus* [26,40,41,42,43,44,45,46,47,48,49,50,51,52], *Staphylococcus epidermidis* [49,53], and *P. aeruginosa* [28,33]. In the current study, we verified the anti-biofilm effect of SPT against *E. coli* ATCC 25922. Significantly, we also demonstrated that SPT can eradicate pre-formed *E.coli* biofilms. As far as we know, this is the first study regarding the employment of SPT against *E. coli*. Previous reports have also pointed out the possible employment of trypsin against *E. coli* biofilms made with milk on stainless steel sheets. However, these researchers employed mechanical disruption with ultrasonication to fully disperse the biofilm [54]. When employing trypsin as a model protease in our study, we did not verify any significant inhibition of *E. coli* biofilm formation. On the other hand, a significant increase was found from 16 to 32 μg/mL of trypsin (Supplementary Figure S1). This result further supports the significance of SPT implementation against *E. coli* biofilms, while also highlighting a possible differential anti-biofilm mechanism when compared with other proteases like trypsin.

The proteolytic activity of SPT has been hypothesized to be the core mechanism of action during biofilm eradication or inhibition, which is an effect targeting not only surface crucial proteins like amyloids, but also intracellular ones [49,55]. However, several studies have challenged the notion of solely proteolytically driven biofilm inhibition [50,52] or introduced the significant effect of SPT on active metabolism, including phosphate homeostasis and NADPH/NADH-related viability [26,27,28]. Indeed, we once again verified that SPT can have a significant effect on the viability of both the planktonic and biofilm bacteria of *E. coli*. Interestingly, this effect does not coincide with reduced planktonic growth, as determined by turbidity measurements, proving that the effect of SPT is not associated per se with toxicity, but is rather a specific anti-biofilm activity. As the MTT reaction is tightly related to NADPH/NADH-dependent oxidoreductases [56], this may indicate that SPT is implicated in some way with the energetic metabolism of the bacteria—an effect verified during the stationary phase, but also possibly present in earlier growth stages, as suggested by the inability of SPT-treated *E. coli* bacteria to adapt to a biofilm phenotype. This effect seems to be crucial for the formation of biofilm, as statistical analysis has verified the correlation between biofilm inhibition and viability. As biofilm formation needs an active metabolism to proceed [17], this may imply the possible implication of SPT on the crucial components of the energetic metabolism of *E. coli*. Indeed, previous studies have indicated that the effect of SPT affects energy metabolism factors in *S. aureus* (pyruvate kinase, transketolase, F1 subunit of ATP synthase, glyceraldehyde 3-phosphate dehydrogenase, etc.) [55]. However, such components or their cofactors are not determined here, and future studies should clarify the exact way SPT is implicated in the energetic metabolism of *E. coli*.

Previously, reduced activity of cellular or secreted ALPs [26,27,28,57] and dysregulated levels of phosphate-binding DING proteins [26] were found to be in conjunction with biofilm dispersion. Given the impact of SPT on bacterial viability under biofilm-forming conditions, ALP activity was studied. In addition, DING proteins, which are known regulators of phosphate metabolism, were determined. While the effect of SPT on cellular ALPs was found to be inconsistent, there was a significant increase in the activity of secreted ALPs. This result is probably related to phosphate starvation. In *E. coli*, the periplasmic ALP is encoded by the *PhoA* [58]—a gene coordinated by the Pho regulon—which controls phosphate homeostasis in bacteria [59]. The activation of the Pho regulon induces an increase in ALPs and DING [59,60,61], while simultaneously inhibiting biofilm formation [62,63,64].

The significance of an active phosphate metabolism in biofilm formation has already been highlighted in studies where the employment of classic ALP inhibitors resulted in biofilm diminishment [27,57]. While increased ALP activity was indeed verified in the extracellular medium, we could not find DING immunoreactivity in the media. Adversely, decreased DING levels were found in the cell lysates, correlating inversely with ALP activity. PstS proteins are among the most canonical bacterial DING proteins. The Pst system participates in the negative regulation of the Pho regulon, since mutations in various genes of the pstSCAB–phoU operon have led to overexpression of Pho-regulated genes, accompanied by the inhibition of biofilm formation. Under phosphate-replete conditions, the repression of the Pho system is mediated by an interaction between the Pst complex and PhoR, which prevents PhoR from phosphorylating PhoB [64]. Although the role of PstS in biofilm formation remains under investigation, the mutation of its gene in *P. aeruginosa* abolishes biofilm development [65], while impaired biofilm formation, coupled with difficulty growing under phosphate-limiting conditions when *pstS* is mutated, has also been observed in *Pseudomonas aureofaciens* [64], *Vibrio cholerae* [66], and *Proteus mirabilis* [67]. In summary, the low levels of DING/PstS detected in *E. coli* under SPT treatment—and their strong correlation with biofilm biomass—suggest a mechanism by which SPT targets PstS, leading, in turn, to Pho induction and ultimately the inhibition of biofilm formation in this bacterium. Indeed, Pho activation is further evidenced by the high alkaline phosphatase activity observed in the presence of SPT.

Curli fibers are regarded as one of the most crucial proteinaceous components of *E. coli* biofilms. Curli biosynthesis is regulated by the csgBA and csgDEFG operons, which encode at least six proteinic co-factors, with CsgB acting as a nucleation factor and membrane anchor, that primes CsgA polymerization [18]. The production of curli fibers is essential for the proper three-dimensional architecture of the extracellular polymeric substance in the biofilm, the vertical growth of bacteria in stacks, and the formation of significant water channels that supply nutrients throughout the entire biofilm volume [68]. The *csgA* and *csgD* genes seem to play a critical role in this process, as their mutation inhibits the ability of *E. coli* to form biofilms, although it allows the formation of small, scattered bacterial aggregates [69], like those observed under the influence of SPT here. The active role of anti-amyloidogenic substances, such as epigallocatechin gallate from green tea, has been shown to be significant in the past for inhibiting biofilm formation by *E. coli*. This is achieved by directly inhibiting the aggregation of curli fibers and interfering with the expression of *csgA*, *csgB*, and *csgD* [70,71]. Additionally, it is important to note that curli mediates the attachment and invasion of *E. coli* on eukaryotic cells [72,73,74], further highlighting the potential benefits of SPT action in vivo.

In this study, we verified the effect of SPT on curli fibers, employing both in vitro and in silico methodologies. Biofilm studies have verified that amyloid/curli fibers are important for consistent biofilm formation and stability, as untreated cultures presented large fibrous, ThT-positive formations. Under the effect of even low SPT doses, these formations are limited in size and disappear altogether by increasing the concentration of the treatment. Previous studies have verified the anti-amyloid potential of SPT, which disrupts the amyloidation of insulin in vitro and in vivo [75,76], Alzheimer’s disease-related amyloid beta 42 [77], and biofilm-related bacterial amyloids [26,28]. In silico results indicate that SPT can directly interact with CsgA and CsgB and possibly block the polymerization of curli fibers, thus explaining the immense effect of the enzyme on the fibrous biofilm formations of *E. coli*. Together, HADDOCK and AlphaFold analyses suggest that both CsgA and CsgB peptides specifically recognize and bind to SPT’s active-site region through extensive, complementary charged interactions. The favorable electrostatic contributions and broad interaction surfaces suggest a robust, specific association that likely facilitates the subsequent proteolysis of curli subunits. This molecular insight supports the potential of SPT as an effective biofilm-disrupting enzyme, prompting the development of future anti-biofilm strategies. This is in agreement with our previous study on the effect of SPT on *P. aeruginosa* biofilm and amyloids, as it was found that SPT can strongly interact with the amyloid peptide FapC, which has an orthologous role in *P. aeruginosa* biofilms [28]. Whether this interaction is proteolytic or not has yet to be defined. In a recent study [52], the authors pointed out that SPT can induce the biofilm inhibition of *S. aureus* via the activity of either the N-terminal catalytic domain (Ncat) or the C-terminal RTX (Repeat-In-Toxin) domain (Crtx), though they also act synergistically for better anti-biofilm capacities. The RTX domain seems to play a distinct, protease-independent role in preventing biofilm formation—potentially in the modulation of cell–surface interactions or signaling pathways. This is also in agreement with Selan et al., who verified that proteolytically inactive SPT retains its anti-biofilm activity against *S. aureus* [50].

The availability of phosphate has been previously shown to exert a profound influence on curli fiber biogenesis in *E. coli* through a rather complex regulatory cascade. Under phosphate-limiting conditions, the PhoBR two-component system activates the Pho regulon and induces the expression of *iraP*, which encodes an anti-adaptor protein that binds to RssB and prevents RpoS delivery to the ClpXP protease, thereby stabilizing σ^S^ (RpoS) during phosphate starvation [78]. The accumulation of RpoS, in turn, promotes the transcription of *mlrA*, which is an MerR-family regulator that directly binds to the *csgD* promoter and upregulates the master biofilm regulator CsgD, leading to enhanced curli subunit (csgBA) expression and fiber assembly [79]. This is consistent with our hypothesis that SPT induces stress from phosphate limitation by impairing Pho regulon activation, as seen in untreated cultures, where extensive fibrous formations were verified; a possible consequence of phosphate limitations that is associated with the stationary phase. Future studies should verify whether the anti-amyloid effect found in this study is due to the dysregulation of phosphate metabolism, and/or a direct inhibitory effect of SPT on Csg isoforms.

Additionally, we found in this study that SPT treatment significantly reduces the levels of both cellular and secreted LPSs. LPSs are critical factors in biofilm formation, constituting 25–30% of its dry weight [80]. Significantly, the structural integrity of the LPSs is essential for *E. coli* virulence in vivo, as the mutation of the O6 antigen in uropathogenic *E. coli* CFT073 leads to the significant attenuation of bladder colonization in a murine urinary tract infection model [81]. In addition, knock-out mutations in chromosomal *waa* loci involved in the biosynthesis of the LPSs’ core oligosaccharide have been shown to limit *E. coli*’s ability to colonize the intestine [82,83]. It is significant that in Gram-negative bacteria, the presence of an LPS-rich outer membrane functions as a selective permeability barrier, effectively excluding macromolecules above ~600 Da unless facilitated by porins or membrane-disrupting agents. This is in contrast to Gram-positive bacteria, where SPT can have direct access to the PG layer and the anchored protein content. This further highlights the significance of our results, as we verified disrupted levels of both LPSs and PG. This could imply a mechanism of bacterial sensitization that would further support the employment of SPT as an alternative antimicrobial agent.

The genes required for LPSs’ synthesis in *E. coli* are induced under stress conditions and associated with enhanced biofilm establishment [84]. Conversely, *waa* variants that produce a more hydrophobic oligosaccharide correspondingly increase membrane hydrophobicity, markedly enhancing biofilm formation and cell aggregation on abiotic surfaces, often accompanied by a loss of flagella [20]. Moreover, a shortened LPSs’ length further reduces the expression of flagellar, fimbrial, and EPS proteins [85]. Phosphorus metabolism in *E. coli*—which is reported to be disrupted by SPT—also contributes to LPSs’ homeostasis. In strains lacking a functional Pst system, there is a significant decrease in lipid A pyrophosphorylation, coupled with the almost complete loss of O-antigen and modification of the core oligosaccharide [86,87]. Such pyrophosphorylation is vital for outer-membrane integrity, cell–cell interactions, antibiotic resistance, and proton permeability. This downregulation aligns with the presence of phosphate-starvation response motifs in LPSs’ biosynthetic genes, making them members of the Pho regulon [88]. Meanwhile, the phosphatase Cdh—potentially responsible for hydrolyzing the lipid A pyrophosphate—was found to be overexpressed, which is a presumed strategy for scavenging phosphate [86]. Together, these data link phosphate homeostasis with LPSs’ synthesis and membrane integrity in Gram-negative bacteria, consistent with the findings of this study.

EPS production requires the formation and secretion of outer-membrane vesicles (OMVs). OMVs carry various PAMPs—such as LPSs, periplasmic proteins, outer-membrane lipids, cytoplasmic proteins, DNA, RNA, and other virulence factors—and their release enhances cell aggregation within biofilms [89]. Certain antibiotics can inhibit cell-wall biosynthesis or trigger OMV formation in response to membrane stress, as part of the bacterial SOS response: a highly conserved mechanism to protect genomic integrity [90]. In this work, SPT treatment reduced *E. coli* PG content and both cellular and secreted LPSs’ levels. Alongside decreased cellular FliC levels, these results suggest a generalized membrane-stress response in *E. coli* under SPT exposure. However, this needs further experimental verification, which was not the scope of this study.

Flagella—and thus FliC—are particularly critical for adhesion to hydrophobic surfaces, as flagellated bacteria adhere more strongly to hydrophobic than hydrophilic substrates [91]. *E. coli* strains lacking *fliC* exhibit reduced motility, biofilm formation, epithelial adhesion, and microcolony development [22,92]. In a murine urinary tract infection model using uropathogenic *E. coli* CFT073, the deletion of FliC markedly attenuated bladder and kidney colonization and prevented dissemination to the spleen 48 h post-inoculation [93]. Conversely, elevated *fliC* expression is crucial during the biofilm dispersal phase, when planktonic cells express higher FliC levels than static-phase cells [94]. Interestingly, curli expression requires initial repression of the FlhDC operon—the master regulator of flagellar genes such as *fliC*. This switch is coordinated by c-di-GMP, which, in the presence of low c-di-GMP, activates the curli regulator CsgD while simultaneously dampening flagellar activity, promoting a biofilm phenotype [95]. Phosphorus metabolism also influences flagellar protein expression: PhoU mutants with constitutive Pho activation display higher levels of flagellar proteins and chemotaxis factors, reflecting an intensified nutrient-seeking effort [96]. In this study, SPT-treated bacteria exhibited both lower amyloid curli and FliC levels, indicating the disruption of the inverse regulation of these factors under phosphate starvation and during transitions between motile and sessile growth. Thus, SPT may impair *E. coli*’s coordinated response to nutrient limitation, aligning with the observed reductions in growth and viability. Whether these effects stem from the decreased transcription of curli and *fliC* or direct proteolytic action of SPT warrants further study.

Despite demonstrating SPT’s potent anti-biofilm activity against *E. coli* and its impact on key biochemical and molecular determinants, our study has several limitations that warrant further investigation. First, the precise mechanism by which SPT perturbs *E. coli* phosphate metabolism remains unresolved; future work should employ targeted molecular assays (e.g., enzyme kinetics and metabolomics) across multiple growth phases to delineate this pathway. Second, the direct influence of SPT on LPSs, FliC, and curli biogenesis requires confirmation through quantitative proteomics and transcriptomic profiling. Third, to substantiate SPT’s proposed anti-virulence properties, in vivo infection models are essential. Moreover, it is unclear whether the observed alterations in phosphate-associated factors reflect a phosphate-specific dysregulation or a broader stress response; this distinction should be addressed in subsequent studies. Finally, our in silico predictions of SPT–CsgA/CsgB interactions must be validated experimentally, and any potential proteolytic or disruptive effects on curli subunits should be characterized in vitro. Collectively, such investigations will be critical to fully elucidate SPT’s mode of action and therapeutic potential.

## 5. Conclusions

In summary, SPT emerges as a potent anti-biofilm agent against *E. coli* by both preventing biofilm formation and eradicating established biofilms—effects that correlate with the possible metabolic dysregulation in planktonic and sessile cells rather than simple growth inhibition. SPT seems to trigger phosphate starvation responses (increased secreted ALP), possibly by downregulating PstS/DING proteins, undermining LPSs’ homeostasis and outer-membrane integrity. Concurrently, it disrupts curli fiber assembly—likely via a direct interaction with CsgA and/or CsgB—and reduces FliC levels, impairing the coordinated switch between motile and sessile states. Together, these multifaceted actions highlight SPT’s promise as a non-antibiotic strategy for both biofilm prevention and removal in medical and industrial settings.

## Data Availability

The raw data supporting the conclusions of this article will be made available by the authors on request.

## Supplementary Materials

The following supporting information can be downloaded at: https://www.mdpi.com/article/10.3390/microorganisms13081875/s1, Supplementary Figure S1. Inhibition of biofilm formation of *Escherichia coli* ATCC 25922 by trypsin. *E. coli* was grown under static conditions in tissue culture plates (TCPs) in the presence or absence of SPT. Biofilm formation was semi-quantified with crystal violet staining after dye extraction and read at 570 nm. Bars represent mean values ± SEM from at least three independent experiments, with the value of the untreated bacteria culture (control) set at 100%. Standard ANOVA with Dunnett’s correction for multiple comparisons was employed for the statistical analysis. Notations for statistically significant differences between control (untreated) and treated samples: * *p* < 0.05; *** *p* < 0.001.
