# Supplementary material for: Serrapeptase Eliminates Escherichia coli Biofilms by Targeting Curli Fibers, Lipopolysaccharides, and Phosphate Metabolism
Source: Microorganisms. 2025 Aug 11;13(8):1875. doi: 10.3390/microorganisms13081875 (PMC12388453; doi:10.3390/microorganisms13081875)
Supplement: Supplementary file 1 [file microorganisms-13-01875-s001.zip › microorganisms-3741161-supplementary.pdf]

SUPPLEMENTARY MATERIAL

Serrapeptase Eliminates *Escherichia coli* Biofilms by Targeting Curli Fibers, Lipopolysaccharides, and Phosphate Metabolism

Georgios Katsipis [1,2], Michalis Aivaliotis [3,4,5] and Anastasia A. Pantazaki [1,2,*]

1 Laboratory of Biochemistry, Department of Chemistry, Aristotle University of Thessaloniki, 54124 Thessaloniki, Greece
2 Laboratory of Neurodegenerative Diseases (LND), Center for Interdisciplinary Research and Innovation (CIRI), Aristotle University of Thessaloniki, 57001 Thessaloniki, Greece
3 Laboratory of Biological Chemistry, School of Medicine, Faculty of Health Sciences, Aristotle University of Thessaloniki, 54124 Thessaloniki, Greece
4 Functional Proteomics and Systems Biology (FunPATh), Center for Interdisciplinary Research and Innovation (CIRI), Aristotle University of Thessaloniki, 57001 Thessaloniki, Greece
5 Basic and Translational Research Unit, Special Unit for Biomedical Research and Education, School of Medicine, Aristotle University of Thessaloniki, 54124 Thessaloniki, Greece
* Correspondence: natasa@chem.auth.gr; Tel: +30-2310-990551

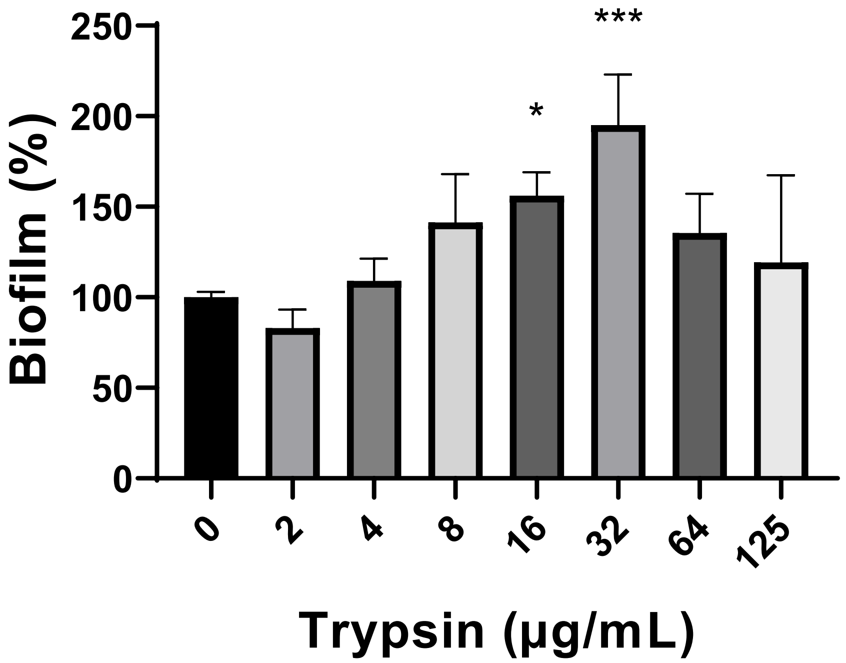

Supplementary Figure S1. Inhibition of biofilm formation of Escherichia coli ATCC 25922 by trypsin. *E. coli* was grown under static conditions in tissue culture plates (TCPs) in the presence or absence of SPT. Biofilm formation was semi-quantified with crystal violet staining after dye extraction and read at 570 nm. Bars represent mean values ± SEM from at least three independent experiments, with the value of the untreated bacteria culture (control) set at 100%. Standard ANOVA with Dunnett's correction for multiple comparisons was employed for the statistical analysis. Notations for statistically significant differences between control (untreated) and treated samples: * $p < 0.05$; *** $p < 0.001$.
